# Has the Rajiv Aarogyasri Community Health Insurance Scheme of Andhra Pradesh Addressed the Educational Divide in Accessing Health Care?

**DOI:** 10.1371/journal.pone.0145707

**Published:** 2016-01-19

**Authors:** Mala Rao, Prabal Vikram Singh, Anuradha Katyal, Amit Samarth, Sofi Bergkvist, Adrian Renton, Gopalakrishnan Netuveli

**Affiliations:** 1 Department of Primary Care and Public Health, Imperial College London, London, United Kingdom; 2 Oxford Policy Management, Delhi, India; 3 ACCESS Health International, Hyderabad, India; 4 SughaVazhvu Healthcare, Thanjavur, India; 5 Institute for Health and Human Development, University of East London, London, United Kingdom; 6 ESRC International Centre for Life Course Studies in Society and Health, University College London, London, United Kingdom; Johns Hopkins Bloomberg School of Public Health, UNITED STATES

## Abstract

**Background:**

Equity of access to healthcare remains a major challenge with families continuing to face financial and non-financial barriers to services. Lack of education has been shown to be a key risk factor for 'catastrophic' health expenditure (CHE), in many countries including India. Consequently, ways to address the education divide need to be explored. We aimed to assess whether the innovative state-funded Rajiv Aarogyasri Community Health Insurance Scheme of Andhra Pradesh state launched in 2007, has achieved equity of access to hospital inpatient care among households with varying levels of education.

**Methods:**

We used the National Sample Survey Organization 2004 survey as our baseline and the same survey design to collect post-intervention data from 8623 households in the state in 2012. Two outcomes, hospitalisation and CHE for inpatient care, were estimated using education as a measure of socio-economic status and transforming levels of education into ridit scores. We derived relative indices of inequality by regressing the outcome measures on education, transformed as a ridit score, using logistic regression models with appropriate weights and accounting for the complex survey design.

**Findings:**

Between 2004 and 2012, there was a 39% reduction in the likelihood of the most educated person being hospitalised compared to the least educated, with reductions observed in all households as well as those that had used the Aarogyasri. For CHE the inequality disappeared in 2012 in both groups. Sub-group analyses by economic status, social groups and rural-urban residence showed a decrease in relative indices of inequality in most groups. Nevertheless, inequalities in hospitalisation and CHE persisted across most groups.

**Conclusion:**

During the time of the Aarogyasri scheme implementation inequalities in access to hospital care were substantially reduced but not eliminated across the education divide. Universal access to education and schemes such as Aarogyasri have the synergistic potential to achieve equity of access to healthcare.

## Introduction

In recognition of the importance of good health to economic and social development, universal health coverage has been placed high on the global political agenda in recent years. Equitable and timely access to health services remain major challenges globally with families in many countries continuing to face financial hardship or even impoverishment as a result of health care bills [1]. Strategies to improve equity of access to health services have, understandably, mainly focused on reducing the financial burden of health care, but studies to examine the determinants of catastrophic health expenditure, i.e. health expenditure which reduces the consumption of necessities below required levels, have demonstrated that non-financial barriers too, can increase its risks [2]. Lack of education has been shown to be a key risk factor for catastrophic health expenditure and consequently, improving educational status needs to be viewed as an important policy instrument that can be used to improve equity of access to health care services [3].

## Background

India has one of the highest levels of out-of-pocket health expenditure [4]. It is also home to the largest number of illiterate people in the world [5]. In response to the need to address the impacts of inequitable access to health care, a new generation of publicly funded government-sponsored health insurance schemes has been launched in recent years, principally aimed at providing financial protection to the poor against catastrophic health shocks, which, for these schemes, the Government has defined as inpatient hospital care [6]. Assessments of their impacts in the early phase, have reported benefits such as reduced out-of-pocket expenditure (OOPE) from some schemes and an increase in the rate of hospitalisations resulting from others [7,8]. But a finding common to several evaluations is that lack of awareness of the schemes and health care entitlements remains a significant barrier to uptake.

India's national *Twelfth Five Year Plan (2012–17)* has proposed that the plethora of health financing schemes which have been implemented across the states should be 'thoroughly studied' so that the lessons could inform an evidence-based roadmap for universal health coverage [9]. This study aimed to assess the impact of the Rajiv Aarogyasri Community Health Insurance Scheme of Andhra Pradesh (AP) which is now 7 years old, in overcoming variations in access to health care due to educational status.

### The Rajiv Aarogyasri Health Insurance Scheme of AP

In 2007 AP launched a pioneering new state-wide fully state-funded health insurance scheme, the Rajiv Aarogyasri Community Health Insurance Scheme (Aarogyasri) to provide treatment for serious and life-threatening illnesses [10]. The specific objectives included: to improve access of poor families to quality 'tertiary' medical care (meaning low-frequency, high cost specialist care) and treatment of identified diseases requiring hospitalisation through an identified network of health care providers, to provide financial cover for catastrophic illnesses which have the potential to wipe out life time savings of poor families and to provide 'universal coverage' to the urban and the rural poor in the state albeit for the conditions covered in the benefits package. All families with a 'below poverty line' (BPL) ration card, i.e. those on an annual income below USD 1384 (INR 75,000) in urban areas and USD 1107 (INR 60,000) in rural areas, and including individuals with pre-existing medical conditions are *automatically* enrolled and the scheme was estimated to cover approximately 20.4 million poor and lower middle class families, comprising about 85 percent of the state's population in 2009 [6]. Enrolees make no contribution; the annual benefit is a maximum of USD 4,500 (INR 200,000) per family per year; and there is no limit on the size of the family [6]. A total of 942 medical and surgical procedures across 31 clinical specialties are provided and the benefits include all inpatient costs—associated investigations, food, transport and medicines for 10 days following discharge. One year follow-up packages including consultation, medicines, and diagnostics are also available for 125 procedures requiring longer periods of follow up. The Aarogyasri scheme has unique features including *Aarogyamithras* (health system navigators), outreach *health camps* delivered by participating hospitals to educate, screen and case-find and a state-of-the-art information technology-based management system. At the time of this study, 353 public and private sector hospitals were 'empanelled' to provide services to Aarogyasri beneficiaries.

In 2009, a descriptive study of the Aarogyasri, based on an analysis of claims data and a survey of beneficiaries, concluded that while the scheme was beginning to reach its intended beneficiaries uptake was lower among scheduled castes and tribes [11]. Fan and colleagues, who used variations in programme roll-out over time and districts to assess the early impacts of the scheme using National Sample Survey data collected before and after its launch, reported significant overall reductions in out-of-pocket expenditure but also found smaller effects on scheduled castes and scheduled tribes households, the most deprived and vulnerable social groups in terms of health and economic status [7]. The authors rightly observed that characteristics such as 'the pervasiveness of discrimination of patients according to their literacy levels and whether they are aware that they are enrolled in the program' are unknown and may partly explain the lower level of impacts in the most deprived groups [7].

## Objectives of the Study

The objective of the study was to examine whether the Aarogyasri scheme has achieved equity of access to hospital inpatient care among households with varying levels of educational attainment.

## Materials and Methods

### Ethical approval

Ethical approval for the study was given by the research ethics committee of the Administrative Staff College of India, Hyderabad, which hosted the study. Household survey questionnaires include signed consent by the head of the household or another adult representative of the household.

### Sampling and data collection

Our study design was based on repeated cross sectional surveys. We used Andhra Pradesh data from the National Sample Survey Organization (NSSO) 2004 decennial Health and Morbidity household survey [12] which pre-dated the launch of the Aarogyasri scheme, as our baseline and used the same survey design to collect post-intervention data in the state in 2012. The survey methodology is described in detail in the 2004 survey report.

The NSSO survey planning ensures equity of representation across the national socio-economic, demographic and geographic landscape. For each state or union territory, samples are divided into two sectors (rural and urban) in proportion to the provisional population as per the most recent Census. The Ministry of Statistics and Programme Implementation of Government of India has a working group including statisticians and economists. This working group is responsible for survey planning including survey design, concepts and definitions, questionnaire design, instructions to the field staff and data collection and analysis for NSSO surveys.

#### First stage sampling—villages and urban blocks

A stratified multi-stage sampling method is used. The first stage units (FSUs) consist of Census villages in the rural sector and Urban Frame Survey (UFS) blocks in the urban sector. The villages in which the survey is to be undertaken are selected by a probability proportional to size with replacement method, from the census listing of villages.

The urban FSUs or UFS blocks are ‘mapped’ by the NSSO, taking into consideration the increase or decrease in the population of urban agglomerations and also newly declared towns, having clear identifiable boundaries and landmarks. Each block includes a population of 600 to 800 people living in approximately 200 households. Every year 20 percent of urban agglomerations are updated and over a period of 5 years, which is known as a ‘phase’, all blocks nationwide are updated. The use of up-to-date maps is therefore essential when replicating the NSS methodology for research.

In our survey carried out in 2012 which replicated the 2004 methodology, we used the ‘First Stage Units’ (FSUs) used by NSSO in their 66th round survey (2008–09) [13], the latest round for which FSUs had been mapped. The FSUs were not identical to those in NSSO 2004, our baseline survey, rapid urbanisation having changed substantially, the urban-rural landscape of the state and thus the geographical basis for sampling units. Our field survey team then carried out the second stage sampling described below.

#### Second Stage Sampling

The second stage sampling is done in the field since no list of households is available. It begins with the selection of hamlet-groups and urban sub-blocks. Firstly, large villages are divided into hamlet-groups and large blocks into sub-blocks. The number of these sub divisions depends on the population of the FSU. The steps followed to delineate these are:

- Step 1: The approximate population of the village is ascertained by seeking the advice of the Panchayat (village council), government school head-teacher or zilla parishad (local government office).
- Step 2: The community groupings or ‘hamlets’ are identified and their geographic boundaries are mapped.
- Step 3: The hamlets are grouped into NSSO ‘hamlet-groups’ for all villages with a population greater than 1200.
- Step 4: Two hamlet groups are randomly selected from these using simple random sampling without replacement and the listing of households is undertaken in these hamlet-groups.

For urban areas the identification of sub-blocks follows roughly similar steps but the sub-blocks are made up of approximately 200 households. Two sub-blocks are selected using simple random sampling without replacement.

The second stage of the sampling focuses on selection of households for the survey. To select the survey households, i.e. the Second Stage Strata (SSS) or USU- Ultimate Sampling Units, households in the selected hamlet-groups and sub-blocks are listed. The selection of 10 households from each hamlet-group or sub-block is by stratified random sampling to ensure that 4 households with at least one member hospitalised during last 12 months, 2 with at least one child below 5 years of age and 2 with at least one member above 60 years of age will be included.

When these data are aggregated at a higher level, representativeness is ensured by multipliers which are to be used as weights. NSSO provides these weights along with the unit level data to be used in statistical analyses.

In summary, 5059 households across all districts of AP were surveyed by the NSSO in 2004. Our survey in 2012 included 8623 randomly selected households representative of all districts and rural and urban areas of the state in that year. The 2012 survey included those households which had benefitted from the Aarogyasri Scheme, as it is operational across the whole state. Household survey questionnaires included signed consent by the head of the household or another adult representative of the household.

#### Primary outcome measures

We used two outcomes, hospitalisation and catastrophic health expenditure for hospital inpatient care. *Hospitalisation* was indicated by a dummy variable where 1 stands for hospitalisation in the past 12 months. Similarly *catastrophic health expenditure* (CHE) was a dummy variable where 1 stands for expenses on hospitalisation which is equal to or more than 10% of total household expenditure, a threshold used in previous studies [14].

*Education*: We used *education* of the head of the household. Education was coded in the surveys as an 11-point ordinal categorical variable with illiteracy at the lowest and post-graduation and above at the highest levels. For the purpose of calculating relative indices of inequality, we transformed this variable into a ridit score. Ridit, transformation was suggested by Bross (1958) for use with ordinal variables and named to highlight its analogy to other transformations like probit and logit [15]. The first three letters refers to “relative to an identified distribution”, to indicate that unlike probit or logit which are based on theoretical distributions, ridit is based on an empirical distribution of a variable in the observed data. The idea of ridit evolved from that of probability transformation and statistical methods using ranks. Ridits are calculated by computing the cumulative frequency of the sample ordered from the lowest to the highest values of the selected variable, here education, and assigning each category with a score equal to the mid-point of the range it occupies on the cumulative distribution.^15^ For example, if 10% of the sample belongs to the highest category, it occupies the range between 0.9 and 1.0 on the cumulative distribution and the ridit score assigned to that category will be the mid-point of this range, (0.9+1.0 = 0.95). As we were interested in comparing the two surveys from 2004 and 2012, we computed the ridit scores using the pooled sample of both surveys. As the data was collected using a complex survey design, we took that also into consideration while calculating the ridit scores.

#### Control variables

We adjusted all analyses for *age and sex*. In addition, we used *income*, *residency and social group*s for stratified analyses. Income, a proxy measure based on household expenditure, was dichotomised at the median as poorer and richer. A dummy variable represented the urban—rural residency status. Social groups were categorised as scheduled castes, scheduled tribes, other excluded groups and all other groups. The 'other excluded groups' referred to the category of 'other backward classes' used by the NSSO [12] (see page 76 of the NSSO 60th Round Report) and in all national reports and documents reporting on social characteristics of the population, for example, the Twelfth Five Year Plan [9].

### Analysis

We used the Kunst-Mackenbach relative index of inequality (RII) suggested by Kunst and Mackenbach [16], which has been used extensively in social epidemiological literature. We refer to this as Kunst-Mackenbach RII to distinguish it from other methods of deriving RII which exist [17]. We chose a relative rather than an absolute measure because our intention was to compare the surveys from 2004 and 2012. An absolute measure would bias the findings if a change in the levels of education occurred without change in the levels of inequality. The RII is computed by regressing the outcome measures on the ridit scores for education using logistic regression models with appropriate weights and accounting for the complex survey design in STATA version 12. The results are odds ratios comparing an individual with the highest qualification with an illiterate individual, adjusted for the distribution of the intermediary levels. Although odds ratios are commonly used as measures of association RIIs are not measuring association but a relative change. A value of RII around one suggests equality and that above one denotes that the educationally advantaged individuals were more likely to be hospitalised or to incur catastrophic health expenditure than the educationally disadvantaged. To study the change in relative inequality, an interaction term between a dummy variable for survey year (2004 = 0, 2012 = 1) and the ridit score for education was created and used in the logistic regression model. For ease of interpretation, we converted it into a percentage change by subtracting one from it and multiplying with 100 as done by others [17]. A positive change means inequalities increased and a negative change means that it decreased.

### Role of the funding sources

The external funding sources had no role in study design, data collection, analysis, interpretation or reporting, or in submission decision.

## Results

### Socio-demographic changes between 2004 and 2012

The population of Andhra Pradesh increased by more than 8.4 million between the two surveys resulting in changes in the socio-demographic characteristics of the two samples. The main changes were increased urbanisation and increased prosperity as denoted by the decreased proportion of the population in the lowest quintile of the assets scale (Table 1). We took account of these differences in our analyses.

**Table 1:** Socio-demographic characteristics of baseline and follow-up samples.

Groups	Andhra Pradesh 2004	Andhra Pradesh 2012
**All**	5059	8623
**Head of Household**		
Male	4433(87.6)	7418(86.0)
Female	626 (12.4)	1205 (14.0)
**Social Group**		
Scheduled Tribes	296 (5.9)	883 (10.2)
Scheduled Castes	974 (19.3)	1797 (20.8)
Other Excluded Groups	2317 (45.8)	3419 (39.7)
All other Groups	1472 (29.1)	2524(29.3)
**Location**		
Rural	3235 (63.9)	4908 (57.0)
Urban	1824 (36.1)	3715(43.0)
**Asset Quintile**		
Lowest	1,594(31.5)	826(9.6)
Second	1,237 (24.5)	1,286(14.9)
Third	753(14.9)	2,121(24.60)
Fourth	744(14.7)	3,072(35.6)
Fifth	730(14.4)	1,318(15.3)

The relative index of inequality compares the person with the lowest educational level with the individual with the highest educational attainment. The first row of Table 2 shows the ridit scores corresponding to the different levels of education. The table also shows the distribution of households in 2004 and 2012 according to education and outcomes: hospitalisation, and catastrophic health expenditure.

**Table 2:** Distribution of households in 2004 and 2012 according to education and outcomes.

	Not literate	Literate- no formal schooling	Below primary	Primary	Middle	Secondary	Higher secondary	Diploma/ certificate	Graduate	Post-graduate and above
Ridit score	0.239334	0.4923125	0.5419035	0.6177766	0.7188612	0.8262795	0.8993243	0.9335768	0.9655755	0.995053
2004										
Number	11172	113	2031	2535	2432	1831	780	168	1123	250
Hospitalisation										
Number	1,059	8	228	272	286	200	87	21	118	29
%	9.5	7.1	11.3	10.8	11.8	10.9	11.2	12.5	10.5	11.6
Catastrophic health expenditure										
Number	4,138	39	870	1,072	1,027	756	316	72	380	102
%	37.0	34.5	42.8	42.3	42.2	41.3	40.5	42.9	33.8	40.8
2012										
Hospitalisation										
Number	1,385	138	145	240	525	445	293	99	169	33
%	9.4	9.5	10.3	9.8	10.3	9.5	9.5	11.5	9.0	9.5
Catastrophic health expenditure										
Number	5,985	525	589	1,059	2,102	1,787	1,185	377	640	139
%	40.7	36.1	41.6	43.4	41.4	38.1	38.4	43.7	33.9	40.2

**Fig 1:**
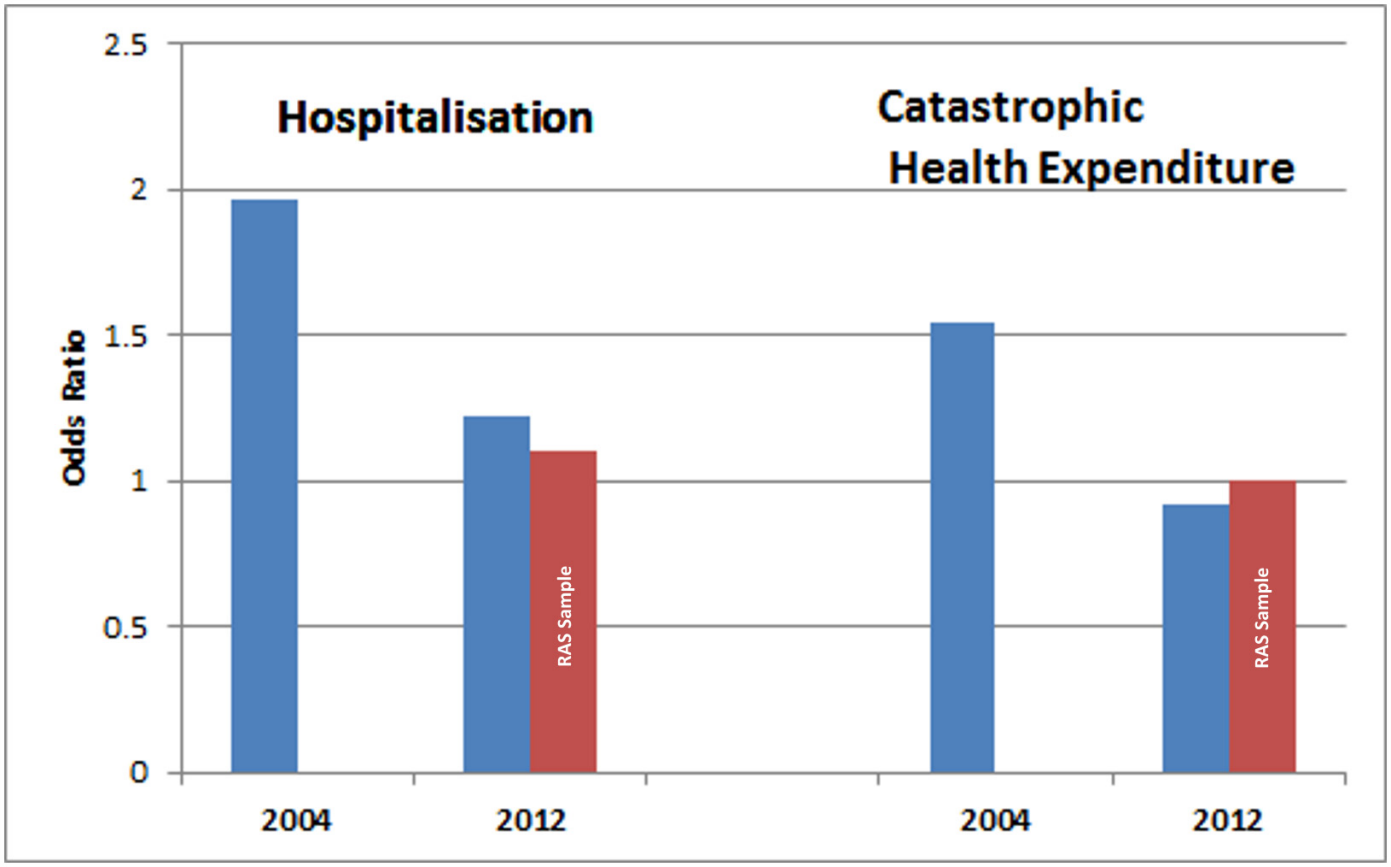
Relative Index of Inequality for hospitalisation and catastrophic health expenditure by education of head of household in 2004 and 2012.

In 2004 the most educated person was twice as likely to be hospitalised as the least educated person (RII 1.97, 95%CI 1.49 to 2.61) in AP (Fig 1). This likelihood was reduced in 2012 (RII 1.22, 95%CI 1.02 to 1.46). In the households that had availed of the Aarogyasri scheme for inpatient hospital care the reduction was more but the RII was not statistically significant (RII 1.13, 95%CI: 0.49 to 2.58). For CHE, the most educated person was one and half times more likely to incur CHE, with a RII of 1.54 (95%CI 1.08 to 2.19) in 2004. In 2012, the inequalities disappeared for the overall samples (RII 0.92 (0.75 to 1.14)) as well as the RAS sample (RII 1.01 (0.17 to 6.11)). Between 2004 and 2012 inequality in hospitalisation reduced by 39% (95%CI -57 to -15) and for CHE by 39% (95%CI -60 to -9) (Tables 3 and 4).

**Table 3:** Relative Index of Inequality (RII) in hospitalisation by education of head of household.

Groups	2004	2012	% Change 2004/2012
	RII (95%CI)	RII (95%CI)	
**Overall**	**1.97 (1.49 to 2.61)**	**1.22 (1.02 to 1.46)**	**-39 (-57 to -15)**
**Subgroups**			
Poorer	**2.06 (1.31 to 3.25)**	1.26 (0.98 to 1.64)	17 (-9 to 51)
Richer	1.26 (0.80 to 1.82)	1.05 (0.81 to 1.35)	-1 (-19 to 20)
Rural	**2.11 (1.37 to 3.23)**	**1.34 (1.06 to 1.71)**	-38 (-62 to 2)
Urban	1.27 (0.81 to 2.01)	1.06 (0.78 1.44)	**-16 (-52 to -45)**
Scheduled castes	1.22 (0.30 to 4.93)	1.06 (0.56 to 2.00)	-17 (-82 to 276)
Scheduled tribes	1.99 (0.96 to 4.14)	1.14 (0.77 to 1.68)	-43 (-75 to 30)
Other excluded groups	**2.89 (1.78 to 4.70)**	1.21 (0.91 to 1.62)	**-59 (-78 to -17)**
All other groups	1.34 (0.88 to 2.04)	**1.47 (1.05 to 2.05)**	7 (-37 to 83)

RII—Relative Index of Inequality. Emboldened numbers represent statistically significant results

**Table 4:** Relative Index of Inequality (RII) in catastrophic health expenditure by education of head of household.

Groups	2004	2012	%Change in RII 2004/2012
	RII (95%CI)	RII (95%CI)	
**Overall**	**1.54 (1.08 to 2.19)**	0.92 (0.75 to 1.14)	**-39 (-60 to -9)**
**Subgroups**			
Poorer	**2.42 (1.25 to 4.66)**	1.02 (0.76 to 1.38)	**-57 (-79 to -12)**
Richer	0.79 (0.53 to 1.17)	0.82 (0.66 to 1.12)	8 (-33 to 78)
Rural	**1.90 (1.07 to 3.35)**	1.02 (0.78 to 1.34)	-45 (-71 to 3)
Urban	0.98 (0.59 to 1.62)	0.83 (0.58 to 1.19)	-16 (-55 to 57)
Scheduled castes	3.00 (0.31 to 28.90)	0.77 (0.37 to 1.61)	-74 (-98 to 178)
Scheduled tribes	1.34 (0.57 to 3.12)	0.93 (0.60 to 1.42)	-28 (-72 to 87)
Other excluded groups	**2.11 (1.18 to 3.78)**	0.89 (0.64 to 1.24)	**-57 (-78 to -17)**
All other groups	0.95 (0.55 to 1.65)	1.11 (0.75 to 1.65)	18 (-40 to 133)

RII: Relative Index of Inequality. Emboldened numbers represent statistically significant results

The results of the sub-group analyses are also presented in Tables 3 and 4. In 2004 inequality in hospitalisation was significantly greater for the poorer (RII 2.06, 95%CI 1.31 to 3.25), rural (RII 2.11, 1.37 to 3.23), and 'other excluded groups' (RII 2.89, 1.78 to 4.70) groups, while in 2012 the inequality was only significant in rural (RII 1.34, 95%CI 1.06 to 1.71) and 'all other groups' (RII 1.47, 95%CI 1.05 to 2.05). Between the two surveys in most groups relative inequality indices decreased but the decrease was significant only in the urban (%change -16, 95%CI -52 to -45) and the 'other excluded groups' (%change -59, 95%CI -78 to -17) groups. For CHE significant RIIs were found only in 2004 poorer (RII 2.42, 95%CI 1.25 to 4.66); rural (RII 1.90, 95%CI 1.07 to 3.35); and 'other excluded groups' (RII 2.11, 95%CI 1.18 to 3.78) groups. As in the case of hospitalisation most of the changes in RIIs between the surveys were to decrease inequality. The two groups in which the changes were significant were poorer (%change -57, 95%CI -79 to -12) and 'other excluded groups' (%change -57, 95%CI -78 to -17) groups.

**Fig 2:**
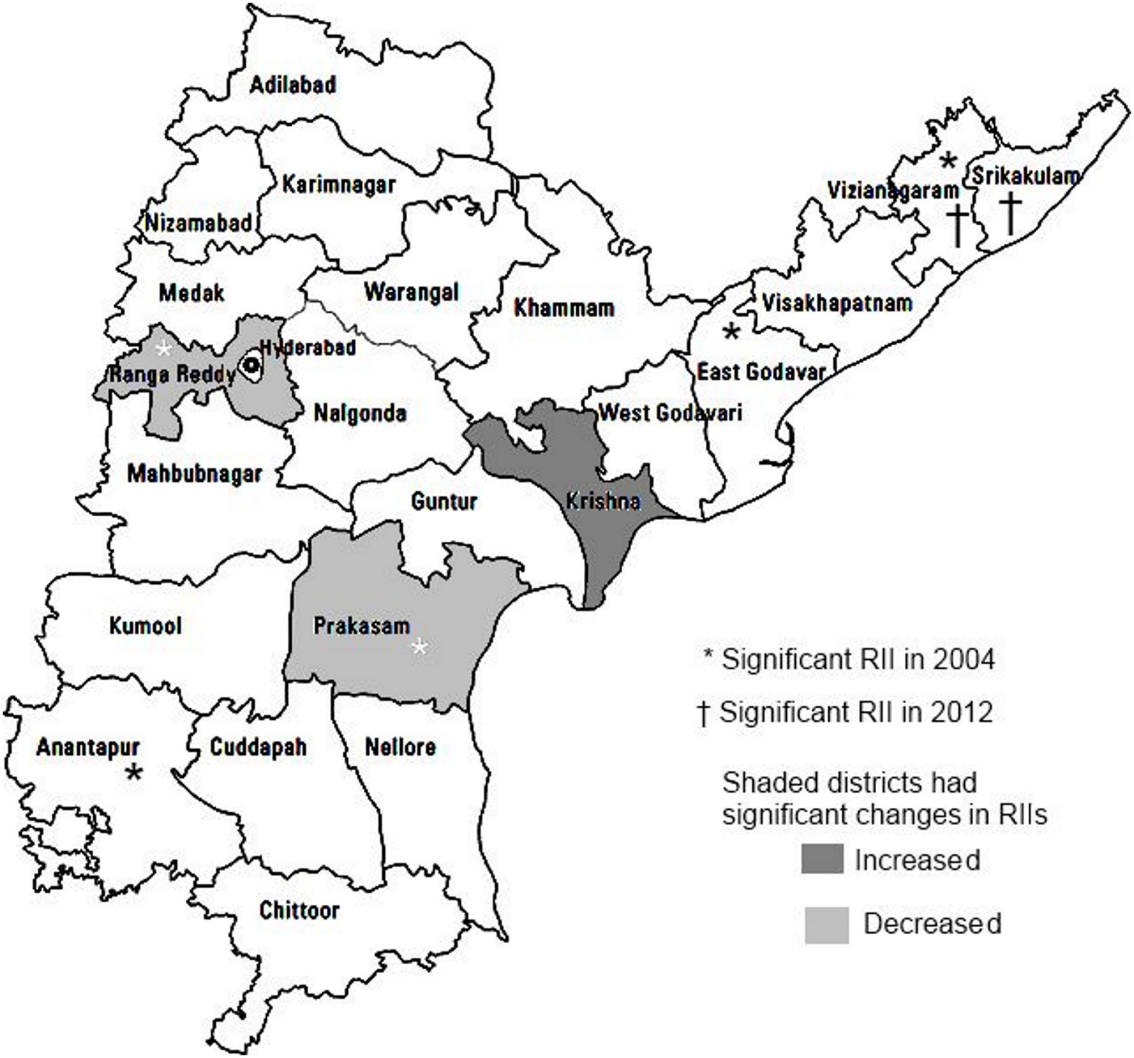
District-wise relative inequality in access to health care by education of head of household in 2004 and change in 2012.

When we compared the relative index of inequality for hospitalisation between districts, we found that inequality indices were significant for five districts in 2004 (Vizianagaram, East Godavari, Rangareddy, Prakasam and Anantapur). (Fig 2). In 2012 only Vizianagaram and Srikakulam had significant RIIs. The most dramatic change was in Rangareddy district where the RII reduced from 20 to 1 and the % change in RII was significant. The only other district with a significant % change was Prakasam. The results for CHE were similar with one exception. In Krishna the relative inequality for CHE increased significantly between 2004 and 2012.

## Discussion

The association between education and health status is well known [18]. Although there is some evidence that lack of awareness, probably resulting from low levels of education is a barrier to health care among the poor, the relationships between educational status, and health care access and expenditure have been less well explored. The objective of this study was to explore whether the Aarogyasri scheme has overcome the educational barrier to access to hospital inpatient care and reducing CHE for inpatient care. Our findings suggest that during the time of the Aarogyasri scheme implementation, equity of access to hospital care has improved across the educational divide, and the dramatic change in Rangareddy district with its proximity to the abundance of high quality tertiary care hospitals in the capital city of Hyderabad may strengthen this conclusion.

Our findings showed that the inequity has also reduced among households which have not used the Aarogyasri scheme. This may be the result of an indirect impact of Aarogyasri which may have resulted in greater competition among health care providers, increasing the availability of 'tertiary' care services and driving down the costs. There are other health initiatives launched in the state during the past decade, the most notable being the National Rural Health Mission aimed principally at improving maternal and child health and the 108 emergency response ambulance service [19, 20]. However, none of these is likely to explain a reduction in inequalities in access to hospital inpatient care and CHE resulting from it.

It is generally assumed that in developing countries an increase in access to hospitalisation is a positive change, and likely to address previously unmet health care needs [21]. Our conclusions are also based on that assumption. However, it is possible that the Aarogyasri scheme has prompted an increase in over diagnosis and unnecessary hospitalisations and treatments by health care providers aware that the costs of care are borne by the state rather than the household. The impact of this would be a doubling of the discrimination endured by households with lower levels of education, unable to access hospital care at times of genuine need, and at other times, becoming victims of over treatment and unnecessary hospitalisation. The state-of-the art IT system and protocol- driven treatment authorisation process integral to the Aarogyasri scheme militates against such negative behaviour, but further study is needed to ensure that the equity of care extends to evidence-based treatments being offered to all households across the educational spectrum [10]. Education is known to have a causal effect on health [18]. Consequently, households with lower levels of education are likely to bear a higher burden of ill health, and an improvement in their access to hospitalisation over time is more likely to suggest that their health needs are being better addressed.

When we consider CHE, RIIs were more than one in all cases where results were significant. This suggests that the more educated segment of the population, who should have better health, had greater catastrophic expenses. This apparent paradox is resolved when we consider that CHE is determined not only by the need for medical treatment but also by the financial capability of the household. The point estimates of the RIIs for the richer, although, non-significant, were all less than one, suggesting they had relatively smaller proportions of households incurring CHE. On the other hand, the better educated among the poorer segment of the population would access healthcare more than the less educated, which will put them at risk of incurring CHE, as our study showed. Importantly, our study also showed that the inequality had almost disappeared by 2012, among those households that had used the Aarogyasri scheme as well as those that had not.

Although we are unable to pinpoint the specific aspect of the scheme to which the reduction in inequality may be attributable, it would be reasonable to speculate that a combination of the non-discriminatory nature of the scheme, near universality of coverage, automatic enrolment into the scheme including those with pre-existing disease with no requirement of payment of premiums, the large number of health care providers in the scheme, and Aarogyamitras appointed at the village and hospital levels to 'hand hold' individuals seeking care and help them navigate themselves through the health system may have achieved this outcome. Further study is needed to determine specific aspects of the scheme to which change may be directly attributable, and which deserve to be replicated across the country.

Further study is also needed to determine the mechanism by which education affects access to health care and lowers the risk of CHE. Possible explanations may be that higher incomes associated with better education directly result in improving access to care and lower the risks of CHE [18]. Better incomes may also be associated with greater hope for the future and a greater likelihood of a household seeking care. Education may improve access to health information and greater confidence in health seeking behaviour and in decisions regarding OOPE. However, our study shows that education alone may not lead to a reduction in CHE, if there is no concomitant improvement in income, but that publicly funded programmes such as Aarogyasri have the opportunity to overcome the impacts of economic inequalities.

Although policies exist to reduce educational inequalities, the pace of change is likely to be slow, and our findings encourage us to suggest that the health delivery system may be able to effect further change in improving health care access for those with lower levels of education, more rapidly. For example, supporting Aarogyasri's ability to reduce inequalities by propping it up on a strong platform of primary care, equipped to provide an effective first point of contact for health care and advice, and to improve health literacy, information, awareness and the confidence of people in making appropriate health care choices may result in further reductions in inequalities more rapidly.

While in general, inequalities by educational status have significantly reduced in the population over time, the reality is that inequalities in access to hospitalisation have persisted across all socio-economic groups. This study confirms that while schemes such as Aarogyasri may substantially reduce such inequalities, achieving equity would require improving levels of education across all socio-economic groups.

In general, evaluations of health financing models have been carried out using a health economics approach. The impact of schemes such as the Aarogyasri is usually not analysed in the context of inequalities. This study is a significant departure from the usual approach. By using an 'inequalities' lens in this analysis, we have taken the issue of universal health care into the arena of social justice. In this study we have demonstrated that the gap between the advantaged and disadvantaged was narrowed, and that this bucks the trend that such programmes usually benefit the better off more [2].

Furthermore, we chose education rather than other ordering measures such as income or assets because of the perceived role of education as the panacea for the multitude of problems India faces. This perception applies to many other developing countries. In addition, economic prosperity is growing in India across the social groups, but it is less clear as to whether educational attainment and literacy are also improving in the most vulnerable communities.

## Limitations of the Study

Analyses of repeated cross sectional surveys aimed at assessing the impacts of interventions, assume that populations demonstrate similar characteristics prior to the start of the intervention, and that 'unobservables' follow a common trend; under such circumstances, any differences in changes observed over time between the population in 2004 and 2012 are attributable to the interventions. But, despite our findings of non-significant differences in changes in the broad socio-economic characteristics of the state, there may have been factors resulting in unobserved differential changes in the populations during the 8 years between 2004 and 2012 to which the results of the analysis may be at least partially attributable.

A second limitation could arise from the impact of other public health programmes implemented during the period 2004 to 2012, despite the fact that the Aarogyasri scheme was the only one aimed at improving access to, and reducing out of pocket expenditure for secondary and tertiary care. The most significant of the other programmes is the National Rural Health Mission launched in 2005, mainly to improve maternal and child health through the revitalization of rural primary care and child and maternal health services. The NRHM is therefore unlikely to have influenced hospital inpatient care.

A further limitation is that a longitudinal study would have provided better evidence for the effectiveness of the scheme. However, in India, as in most developing countries, such longitudinal data are not available through cohort or panel surveys. Also the evaluation of policies or programmes is usually not built in when such policies and programmes are implemented because many of them are politically motivated, as is the case with the Rajiv Aarogyasri scheme. Evaluation is often considered as an afterthought or in response to some need that arises later. However, we were fortunate to have the NSSO survey in 2004 to use as a baseline and we took care that our follow up survey closely followed its design so that together they constituted a comparable pair of repeated cross-sectional surveys. Even in countries such as the UK with its wealth of panel, longitudinal and cohort studies, repeated surveys like Health Surveys for England and Scotland are often used to study the impact of policy changes.

This study has examined health related expenditure and behaviours at only two points in time. To establish trends, more than two time points are needed. However, it needs to be recognised that schemes such as the Aarogyasri themselves may not have longevity and may change or be replaced in response to changing needs. King et al acknowledged that their assessment of the Mexican Seguro Popular programme (at 10 months) published in the Lancet in 2009 [22], was undertaken at an early stage, but it was nevertheless recognised as having provided important evidence of impacts, albeit early ones. Therefore a realistic aim of an evaluation in this rapidly changing health delivery landscape in India would be to study change over time, even if that is limited initially to two time points, but to continue to evaluate the system repeatedly, and use the evidence to reshape health delivery to be responsive to future socio-economic and epidemiological trends.

Another limitation is that our results are 'coarse grained' because the smallest comparable units between two time points in our study were the districts. Therefore, we describe marginal changes at the district level. However, using analyses suitable for complex surveys we provide results that are useful for the policy maker. We have provided data supporting the comparability of the districts at the two time points.

## Conclusion

The international literature on the impact of health financing schemes highlights literacy as a barrier, but does not explore further the relationship between education and access to health care. Our study has shown that universal access to education and refining the state sponsored Aarogyasri scheme to further improve health literacy have the synergistic potential to achieve social justice through equality of access to health care and reduced health expenditure in the future.
